# Supplementary material for: Outpatient Department Visits and Mortality with Various Causes Attributable to Ambient Air Pollution in the Eastern Economic Corridor of Thailand
Source: Int J Environ Res Public Health. 2022 Jun 23;19(13):7683. doi: 10.3390/ijerph19137683 (PMC9265572; doi:10.3390/ijerph19137683)
Supplement: Supplementary file 1 [file ijerph-19-07683-s001.zip › ijerph-1746851-supplementary.pdf]

Supplementary materials

Outpatient Department Visits and Mortality with Various Causes Attributable to Ambient Air Pollution in the Eastern Economic Corridor of Thailand

Khanut Thongphunchung [1], Panita Charoensuk [1], Sutida U-tapan [1], Wassana Loonsamrong [1], Arthit Phosri [2,3] and Wiriya Mahikul [4,*]

[1] Health Impact Assessment Division, Department of Health, Ministry of Public Health, Nonthaburi 11000, Thailand; khanut.th@gmail.com (K.T.); panita.charoensuk@gmail.com (P.C.); usutida@gmail.com (S.U.-t.); loonsamrong.blue@gmail.com (W.L.)

[2] Department of Environmental Health Sciences, Faculty of Public Health, Mahidol University, Bangkok 10400, Thailand; arthit.pho@mahidol.ac.th

[3] Center of Excellence on Environmental Health and Toxicology, Bangkok 10400, Thailand

[4] Princess Srisavangavadhana College of Medicine, Chulabhorn Royal Academy, Bangkok 10210, Thailand

* Correspondence: wiriya.mah@cra.ac.th; Tel.: +66-93194-2944

Supplementary materials

Table S1 Associations between unit increase in pollutant concentrations and outpatient department visits for specific causes.

Table S2 Associations between unit increase in pollutant concentrations and mortality for specific causes.

Table S1 Associations between unit increase in pollutant concentrations and outpatient department visits for specific causes.

Diagnostic category	Coefficient (Standard Error) per unit increase in concentration					
	PM_{10} (μg/m³)	$PM_{2.5}$ (μg/m³)	NO_2 (ppb)	SO_2 (ppb)	O_3 (ppb)	CO (ppm)
**Circulatory diseases**	0.002198 (0.000611)	-0.000100 (0.001097)	0.013706 (0.001711)	0.005982 (0.004158)	-0.000200 (0.000561)	0.033048 (0.035512)
Pulmonary heart disease and diseases of pulmonary circulation	0.002497 (0.000738)	0.000900 (0.001300)	0.013607 (0.002038)	0.007075 (0.005066)	0.000100 (0.000689)	0.055624 (0.041964)
**Respiratory diseases**	0.002098 (0.000382)	0.000300 (0.000663)	0.009257 (0.001036)	0.012225 (0.002545)	0.000600 (0.000357)	0.034401 (0.021757)
Acute upper respiratory infections	0.002198 (0.000382)	0.000200 (0.000638)	0.009158 (0.001062)	0.014396 (0.002515)	0.000800 (0.000357)	0.035753 (0.021728)
Influenza and pneumonia	0.001898 (0.000586)	0.001199 (0.001198)	0.009158 (0.001744)	0.002098 (0.004277)	0.000500 (0.000535)	0.049552 (0.035440)
Acute lower respiratory infections	0.001599 (0.000458)	0.000800 (0.000943)	0.007174 (0.001342)	0.010247 (0.003182)	-0.000300 (0.000434)	0.021664 (0.027122)
Diseases of upper respiratory tract	0.002397 (0.000611)	0.000500 (0.001224)	0.012719 (0.001788)	0.012126 (0.004410)	-0.000100 (0.000587)	0.052403 (0.037464)
Chronic lower respiratory diseases	0.001499 (0.000509)	0.000500 (0.001045)	0.010445 (0.001464)	0.001898 (0.003539)	0.000000 (0.000485)	0.016365 (0.030549)
**Skin and subcutaneous tissue diseases**	0.001798 (0.000407)	0.000500 (0.000688)	0.010247 (0.001212)	0.007075 (0.002837)	0.000000 (0.000408)	0.042389 (0.025053)
Dermatitis and eczema	0.002198 (0.000484)	0.000700 (0.000790)	0.012027 (0.001386)	0.007571 (0.003215)	0.000100 (0.000434)	0.050693 (0.028322)

Table S2 Associations between unit increase in pollutant concentrations and mortality for specific causes.

Diagnostic category	Coefficient (Standard Error) per unit increase in concentration					
	**PM_{10} (μg/m³)**	**$PM_{2.5}$ (μg/m³)**	**NO_2 (ppb)**	**SO_2 (ppb)**	**O_3 (ppb)**	**CO (ppm)**
**Circulatory diseases**	0.002228 (0.001502)	0.007472 (0.002785)	0.011632 (0.004564)	0.015381 (0.010852)	0.002696 (0.001399)	0.269722 (0.094227)
Pulmonary heart disease and diseases of pulmonary circulation	0.003494 (0.002669)	0.015480 (0.005877)	0.011138 (0.008174)	0.024985 (0.019650)	-0.002202 (0.002480)	0.285104 (0.177841)
**Respiratory diseases**	0.001599 (0.001987)	0.008167 (0.004074)	0.009257 (0.006167)	-0.007629 (0.014547)	0.000600 (0.001836)	0.064851 (0.131337)
Acute upper respiratory infections	-0.032951 (0.005702)	0.058740 (0.018085)	0.103729 (0.027285)	0.209694 (0.047670)	-0.006723 (0.004546)	NA
Influenza and pneumonia	0.002397 (0.002443)	0.013212 (0.005136)	0.015283 (0.007511)	-0.025523 (0.018890)	-0.000100 (0.002271)	0.200816 (0.163028)
Acute lower respiratory infections	0.091302 (0.009220)	0.046120 (0.014468)	0.055813 (0.029996)	0.415019 (0.060299)	0.008762 (0.006954)	NA
Diseases of upper respiratory tract	0.071017 (0.010407)	NA	-0.005917 (0.020343)	0.070086 (0.036146)	-0.009545 (0.008087)	NA
Chronic lower respiratory diseases	-0.001301 (0.003550)	0.019607 (0.008730)	0.004490 (0.011046)	-0.018368 (0.028228)	0.001898 (0.003157)	-0.237242 (0.243472)
**Skin and subcutaneous tissue diseases**	0.007869 (0.003847)	0.028685 (0.009593)	0.050883 (0.011806)	0.027712 (0.029164)	0.001399 (0.003414)	-0.063046 (0.328499)
Dermatitis and eczema	0.025375 (0.010644)	-0.012984 (0.081326)	0.189463 (0.054376)	NA	NA	NA

Note: NA = no data available.
